# Biogenerated Oxygen‐Related Environmental Stressed Apoptotic Vesicle Targets Endothelial Cells

**DOI:** 10.1002/advs.202306555

**Published:** 2024-03-13

**Authors:** Qiuyu Zhao, Bolun Lu, Shutong Qian, Jiayi Mao, Liucheng Zhang, Yuguang Zhang, Xiyuan Mao, Wenguo Cui, Xiaoming Sun

**Affiliations:** ^1^ Department of Plastic and Reconstructive Surgery Shanghai Ninth People's Hospital Shanghai Jiao Tong University School of Medicine 639 Zhi Zao Ju Road Shanghai 200011 P. R. China; ^2^ Department of Plastic Surgery The First Affiliated Hospital College of Medicine Zhejiang University Hangzhou 310003 P. R. China; ^3^ Department of Orthopaedics Shanghai Key Laboratory for Prevention and Treatment of Bone and Joint Diseases Shanghai Institute of Traumatology and Orthopaedics Ruijin Hospital Shanghai Jiao Tong University School of Medicine 197 Ruijin 2nd Road Shanghai 200025 P. R. China

**Keywords:** angiogenesis, apoptotic vesicles, microspheres, oxygen‐related stress

## Abstract

The dynamic balance between hypoxia and oxidative stress constitutes the oxygen‐related microenvironment in injured tissues. Due to variability, oxygen homeostasis is usually not a therapeutic target for injured tissues. It is found that when administered intravenously, mesenchymal stem cells (MSCs) and in vitro induced apoptotic vesicles (ApoVs) exhibit similar apoptotic markers in the wound microenvironment where hypoxia and oxidative stress co‐existed, but MSCs exhibited better effects in promoting angiogenesis and wound healing. The derivation pathway of ApoVs by inducing hypoxia or oxidative stress in MSCs to simulate oxygen homeostasis in injured tissues is improved. Two types of oxygen‐related environmental stressed ApoVs are identified that directly target endothelial cells (ECs) for the accurate regulation of vascularization. Compared to normoxic and hypoxic ones, oxidatively stressed ApoVs (Oxi‐ApoVs) showed the strongest tube formation capacity. Different oxygen‐stressed ApoVs deliver similar miRNAs, which leads to the broad upregulation of EC phosphokinase activity. Finally, local delivery of Oxi‐ApoVs‐loaded hydrogel microspheres promotes wound healing. Oxi‐ApoV‐loaded microspheres achieve controlled ApoV release, targeting ECs by reducing the consumption of inflammatory cells and adapting to the proliferative phase of wound healing. Thus, the biogenerated apoptotic vesicles responding to oxygen‐related environmental stress can target ECs to promote vascularization.

## Graphical abstract

Apoptotic vesicles of MSCs prestimulated by hypoxia or oxidative stress are fabricated inspired by the wound microenvironment. These two oxygen‐related environmental stressed ApoVs can deliver similar miRNA cargoes to promote angiogenesis. In this process, the miRNA‐210/Akt axis contributes to endothelial cell migration.

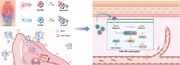

## 1 Introduction

Hypoxia and oxidative stress are widely associated with the onset and progression of microvascular dysfunction‐related diseases such as diabetic microvascular complications, chronic wounds, and cancer.^[^ ^1^ ^]^ Importantly hypoxia and oxidative stress coexist and crosstalk at the intracellular level, and both contribute to oxygen‐related environmental stress in the pathological microenvironment. In biological processes, the local oxygen level alters the cellular cargoes continuously exchanged in intercellular communication networks.^[^ ^2^ ^]^ Thus, oxygen‐related environmental stress triggers a multidimensional and complex regulatory network in intracellular, intercellular, and local microenvironments, which contribute to pathophysiological conditions. Previous studies have aimed to explore separate targeting strategies for hypoxia or oxidative stress in disease;^[^ ^3^ ^]^ however, this is not compatible with the complicated pathophysiological environment in vivo. The interplay between hypoxia and oxidative stress has also been documented. Cao et al. found that the interaction between two typical proteins, Thioredoxin and hypoxia‐inducible factor 2α, involved in the hypoxia and oxidative stress pathways promotes hepatocellular carcinoma metastasis.^[^ ^4^ ^]^ However, the mechanism underlying the crosstalk between hypoxia and oxidative stress in damaged tissue regeneration remains elusive.

Several extracellular vesicles (EVs) are involved in intercellular communication. Exosomes, ectosomes, microvesicles, membrane vesicles, and apoptotic vesicles (ApoVs) can be distinguished by their biogenetic pathways.^[^ ^5^ ^]^ Among the vesicles, ApoVs are produced in an exceptionally simple manner. As most EVs are derived from the paracrine pathway, ApoVs are products of disassembled apoptotic cells with large particle sizes and characteristic surface markers of apoptotic pathways.^[^ ^6^ ^]^ Therefore, ApoVs are often used as engineered delivery vehicles. For example, Dou et al. constructed chimeric apoptotic bodies (cABs) functionalized with apoptotic body membranes and mesoporous silica nanoparticles (MSNs) to regulate inflammation.^[^ ^7^ ^]^ However, these cABs are different from Abs in terms of contents and therapeutic effects. In addition, ApoVs, from the apoptotic pathway, are the dominant therapeutic mediators in systemic cell transplantation as it is hard for cell grafts to survive and sustain paracrine function in the complex microenvironment in vivo,^[^ ^6^ ^]^ which is subject to local inflammation, oxygen‐related environmental stress, and filtration by the microvascular system. However, transplanted cells with extensive apoptosis can still provide durable therapeutic effects.^[^ ^8^ ^]^ Microenvironmental stimulation of the parent cells is reflected in the cargo composition of ApoVs. Until now, studies have mainly focused on ApoVs produced by normal cells; however, the mechanisms of ApoVs generated in specific environments remain largely unknown. Consequently, ApoVs could reveal the regulatory mechanisms involved in oxygen‐related environmental stress.

In the pathophysiology of tissue repair, the combination of hypoxia and oxidative stress leads to non‐healing.^[^ ^9^ ^]^ Angiogenesis is the basis of tissue repair. Nutrients and oxygen are supplied and metabolites are removed by the vascular bed to support cell proliferation and collagen deposition at the site of injury for the healing process.^[^ ^10^ ^]^ Endothelial cells (ECs) are the main cells of the microvessels involved in oxygen delivery to the local tissues. Stimulation of oxygen‐related environmental stress regulatory networks makes endothelial cells a target for improving tissue adaptation to abnormal oxygen microenvironments. Mesenchymal stem cells (MSCs) are pluripotent stem cells with strong self‐renewal, multidirectional differentiation, and paracrine capacity. Both MSCs and their cellular products have been considered effective vehicles for immunomodulation and injury repair.^[^ ^11^ ^]^ MSC‐derived apoptotic vesicles (MSC‐ApoVs) have been proven to play a role in many repair‐related conditions, especially immune regulation.^[^ ^6^ ^]^ The potential of stem cell‐derived ApoVs for angiogenesis has been implied. Our previous studies have demonstrated that nanovesicles fabricated from MSC‐AB membranes promote vascularization by biotargeting ECs for drug delivery through a “Find‐Eat” strategy.^[^ ^12^ ^]^ However, the effects of intercellular signal molecules present in ApoV cargo on angiogenesis have not yet been fully elucidated under the specific microenvironment targeting endothelial cells.

**Scheme 1:**
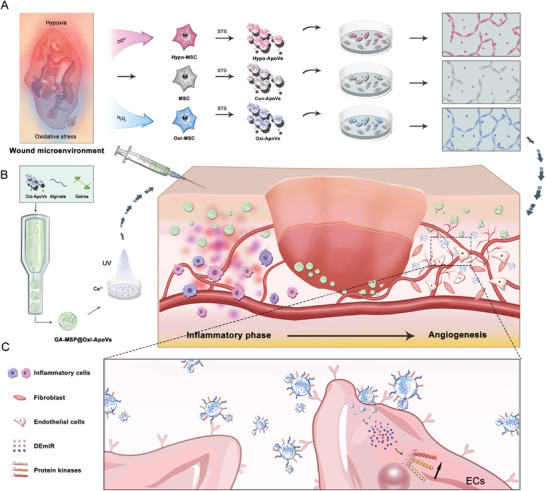
A) Inspired by the microenvironment of injured tissues, ApoV obtained from MSCs treated in an oxygen‐related stress environment better promotes endothelial cell angiogenesis. B) Microspheres loaded with oxidative stress‐derived ApoVs (GA‐MSP@Oxi‐ApoVs) were prepared by microfluidic technology, and injected in situ at the site of injured tissues adapting to the proliferative phase to release Oxi‐ApoVs and promote wound healing. C) Mechanisms of ApoVs promoting endothelial cell function. ApoVs stimulated by the oxygen stress environment are specifically taken up by endothelial cells, in which altered miRNA profiles and upregulate endothelial cell protein kinases.

Therefore, we intended to investigate the therapeutic effects of ApoV biogenerated under oxygen‐related environmental stress targeting endothelial cells for vascularization and to clarify the mechanisms and the similarities and differences between the two types of ApoV. In this study, it is hypothesized that the miRNAs commonly upregulated in oxygen‐related environmental stressed MSC‐ApoVs are the key factors for the enhanced therapeutic effect. First, we propose that systemically administered MSCs and chemically induced ApoVs have similar therapeutic effects in a wound model; however, in vivo oxygen‐related environmental stress and apoptotic experience of MSCs further enhance angiogenesis and promote injury healing. Thus, two oxygen‐related environmental stressed MSC‐ApoV are biogenerated by chemical‐induced apoptosis of MSCs after exposure to oxygen‐related environmental stress using cobalt chloride or hydrogen peroxide. Subsequently, based on the complex interactions between hypoxia and oxidative stress, we compared the similarities and differences in the effects of two types of ApoVs on recipient cells in vitro. As a result, oxidative stress ApoVs (Oxi‐ApoVs) have the strongest biological effects compared to hypoxic and normoxic ApoVs. Then the underlying molecular mechanisms and common pathways were investigated. The two oxygen‐related environmental stressed ApoVs shared similar miRNA profiles and promoted EC phosphokinase activation, with the miR‐210/Akt pathway being responsible for promoting endothelial cell migration. Finally, we designed a GelMA‐alginate interpenetrating hydrogel microsphere (*GA‐MSP*) for local delivery with in vivo MSC paracrine mimetic properties, capable of reducing early inflammatory cell consumption of ApoVs by controlled release, adapting to the proliferative phase of wound healing and achieving long‐term retention of Oxi‐ApoVs, targeting ECs in a diabetic chronic wound model (**Scheme** **1**).

## 2 Results and Discussion

### 2.1 MSC‐ApoVs and MSC Transplants Have Similar Therapeutic Outcomes

**Figure 1:**
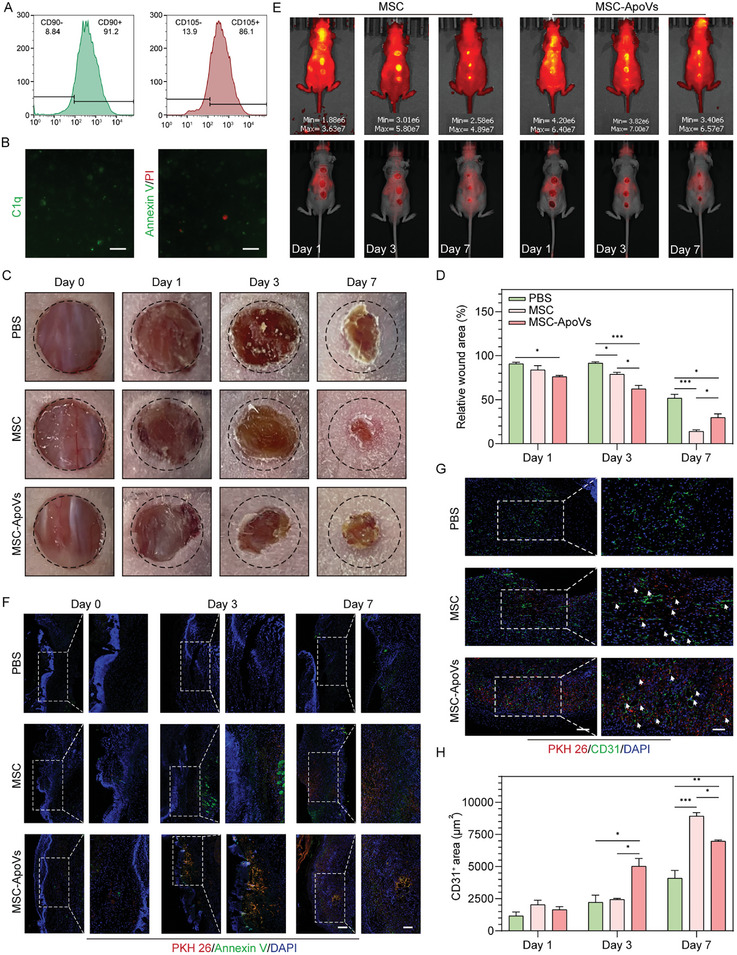
Intravenous MSCs or MSC‐derived ApoVs localized to the skin injury area in nude mice to promote wound healing. A) Flow cytometry results of MSC markers CD90 and CD105 inherited from MSC‐ApoVs. B) Fluorescence staining images of apoptosis markers Annexin V/PI and C1q on the surface of ApoVs. Scale bar  =  10 µm. C,D) Representative images of each group of wounds at different time points and wound closure rate. E) Fluorescence images of the dorsal skin of mice intravenously injected with DiD‐labeled MSCs or MSC‐ApoVs. F) Localization of apoptotic products of MSC or ApoVs in wounds. Left: scale bar  =  100 µm, right: scale bar  =  50 µm. G,H) Co‐localization of PKH26‐labeled MSC apoptotic products or ApoVs with neovascularization and neovascularization quantification. Left: scale bar  =  100 µm, right: scale bar  =  50 µm. ^*^ *p* < 0.05, ^**^ *p* < 0.01, ^***^ *p* < 0.001 (*n* = 3, biological replicates per group).

MSC apoptosis was induced by staurosporine for 12 h. The vesicular structures formed by MSC apoptosis were observed under an inverted microscope (Figure S1A, Supporting information). By immunofluorescence staining, we found that ApoVs inherited the parental surface markers CD90/CD105 (**Figure** **1**A) and expressed the apoptosis‐related marker, C1q, and phosphatidylserine (PS) that binds Annexin V (Figure 1B). The phase of apoptosis was additionally identified by flow cytometry with Annexin V/PI double staining. We found that 73.5% of ApoVs were in the early apoptosis phase with undisrupted membrane integrity and permeability (Figure S1B, Supporting information).

The pluripotency of MSCs has led to the belief that these cells assisted in host regeneration by differentiation and direct replacement of injured cells. Later, it was found that the function of transplanted MSCs is mediated via the secretome. There is growing evidence that transplanted MSCs remain viable transiently in the recipient, after which the cells undergo apoptosis in the host circulation or the transplanted tissue.^[^ ^6^, ^8^ ^]^ For the preliminary validation of the vasculogenic effect of apoptotic vesicles, a nude mouse skin wound model was constructed, and MSCs or MSC‐ApoVs were administered by tail vein injection. Compared to the PBS group, both experimental groups showed enhanced wound healing on day 7. While MSC‐ApoVs group showed a higher healing rate than the MSC group on day 3, the wound area was the smallest in the MSC group on day 7 (Figure 1C,D). Though HE staining showed shorter scar lengths, greater epidermal thickness, and more peri‐wound inflammatory cell aggregation in MSC‐ApoVs group than in the PBS group on day 7, the MSC group had the best healing with mild inflammatory response, and new skin appendages formation(Figure S1C, Supporting information).

In vivo, fluorescence imaging showed that MSCs and MSC‐ApoVs were detectable on the dorsal skin 1 day after dosing and were progressively enriched until day 7 (Figure 1E). Previous studies have shown that MSCs first assemble in the lungs before redistributing and undergoing rapid and extensive apoptosis, regardless of local or systemic administration.^[^ ^13^ ^]^ The frontal view revealed that MSCs had decondensed from the lungs after the first 24 h, and therefore artifacts due to the pulmonary distribution can be excluded (Figure S1D, Supporting information). In order to explore the distribution, condition, and therapeutic effect of MSCs and MSC‐ApoVs gathered in the wound, we labeled MSCs and MSC‐ApoVs with red PKH26 and simultaneously stained tissue sections with the immunofluorescent Annexin V and CD31. Figure 1F shows the performance of the injectant in the wound tissue over time. In the first 3 days, only a small number of red‐labeled cells were observed and co‐stained with green Annexin V, suggesting that MSCs underwent apoptosis in vivo and gradually migrated to the peri‐wound area; a large number of apoptotic MSCs were observed on day 7. Interestingly, MSC‐ApoVs were clearly detectable on day 3, persisting until day 7. Endothelial marker, CD31 staining showed that both apoptotic MSC products and MSC‐ApoVs were phagocytosed by ECs on day 7 (Figure 1G). The CD31^+^ area confirmed the role of MSCs or MSC‐ApoVs (Figure 1H). MSC‐ApoV injection resulted in the highest number of neovessels on day 3. On day 7, the most significant angiogenesis occurred in the MSC group. These results demonstrate that MSC‐ApoVs act in the early stages of wound healing, in comparison to MSC grafts which undergo apoptosis in vivo, thereby improving the outcome of injured tissue repair and angiogenesis. We suggest that two factors lead to this phenomenon. The first is the timing of ApoV release; apoptotic MSCs appeared at a later stage, indicating that better wound healing requires a slow release of ApoVs. Second, microcirculatory disturbances during injury repair lead to hypoxia and high levels of ROS in local tissues (Figure S1E, Supporting information), which may alter the biological synthesis of MSCs, in turn affecting the biological signals transmitted by MSC‐ApoVs in vivo, which promotes angiogenesis after being phagocytosed by ECs. Based on these assumptions, we constructed oxygen‐related environmental stressed MSC‐ApoVs and investigated the effective characteristic composition and mechanism of angiogenesis.

### 2.2 ECs are Activated by MSC‐ApoVs From Different Oxygen Environments

**Figure 2:**
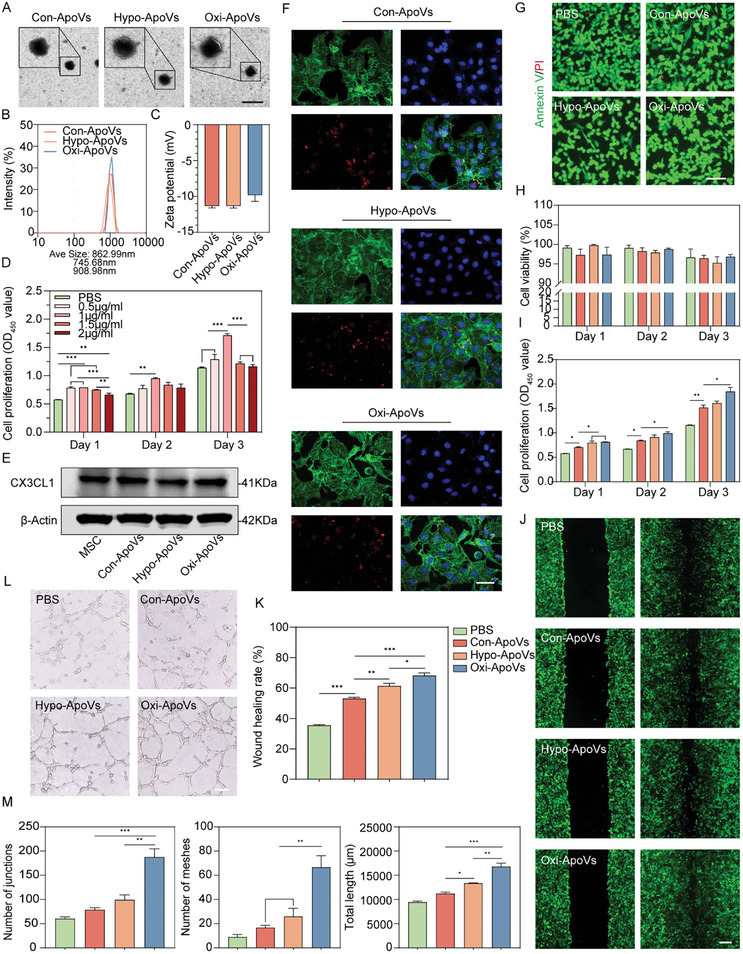
Oxygen‐related environmental stressed ApoVs fabricated in vitro enhance ECs functions. A) TEM image of Con‐ApoVs, Hypo‐ApoVs and Oxi‐ApoVs. Scale bar  =  2 µm. B,C) Diameter and zeta potential measurement. D) Effect of different concentrations of Con‐ApoVs on the EC proliferation. E) Protein expression analysis of CX3CL1. F) Typical fluorescence image of ECs cocultured with Con‐ApoVs, Hypo‐ApoVs, and Oxi‐ApoVs after 6 h. Scale bar  =  50 µm. G,H) Images of live/dead staining and cell viability of ECs cocultured with each group of ApoVs at the optimal concentration. Scale bar  =  100 µm. I) The effect of Con‐ApoVs, Hypo‐ApoVs, and Oxi‐ApoVs on ECs proliferation. Scale bar  =  100 µm. J,K) Wound healing images and quantitative analysis of ECs after 24 h of treatment with each group of ApoVs. Scale bar  =  100 µm. L,M) Images and quantitative analysis of tube formation experiments. Scale bar  =  100 µm. ^*^ *p* < 0.05, ^**^ *p* < 0.01, ^***^ *p* < 0.001 (*n* = 3, biological replicates for each group).

The abnormal oxygen microenvironment affects the biological signals and functions of MSCs and their products.^[^ ^14^ ^]^ Therefore, we investigated whether oxygen‐related environmental stressed ApoVs generated from pre‐treated MSCs that were subjected to hypoxia or oxidative stress in vitro would target ECs and promote angiogenesis. According to the reference, MSCs were cultured in a simulated hypoxic (100 µm CoCl_2_·6H2O)^[^ ^15^ ^]^ or oxidative stress (50 µm H_2_O_2_)^[^ ^16^ ^]^ environment. Next, chemical apoptosis was induced. Then, control apoptotic vesicles (Con‐ApoVs) from the normoxic environment, hypoxia apoptotic vesicles (Hypo‐ApoVs), and oxidative stress apoptotic vesicles (Oxi‐ApoVs) were collected. Transmission electron microscope (TEM) and Dynamic light scattering (DLS) showed that all ApoV types were spherical‐like vesicles (**Figure** **2**A) with particle sizes between 600 and 1200 nm (Figure 2B). The zeta potentials of all three ApoVs were ≈−10 mV (Figure 2C), indicating good colloidal stability of the ApoVs, which implies less ApoV disruption and fewer protein impurities.^[^ ^17^ ^]^ It is consistent with the results of previous Annexin V/PI flow cytometry experiments. Overall, the results indicate that there is no significant difference in the characteristics of different types of oxygen‐stressed MSC‐ApoVs.

ECs were then treated with varied concentrations of Con‐ApoVs to confirm a safe range. Live/dead staining showed a significant decrease in cell viability when Con‐ApoVs concentrations were present at concentrations higher than 2.5 µg mL^−1^ (Figure S2A,B, Supporting information), indicating that high concentrations of ApoVs are cytotoxic. The optimum concentration was determined using the CCK8 cell viability assay within the safe range, and it was found that EC viability increased significantly at 1 µg mL^−1^ Con‐ApoVs (Figure 2D). Annexin V was used to observe PS exposure on the membrane after apoptosis, which is a nonspecific “eat‐me” signal on the surface of the ApoVs. CX3CL1 is a chemokine that specifically binds to PS, and CX3CL1/CX3CR1 has been considered a specific receptor/ligand that mediates MSC‐ApoVs phagocytosis.^[^ ^12^, ^18^ ^]^ Therefore, we assessed CX3CL1 expression in MSCs and each group of ApoVs via western blotting. CX3CL1 was expressed in all groups but was slightly down‐regulated in Hypo‐ApoVs (Figure 2E; Figure S2C, Supporting information). The ECs were then cocultured with different types of ApoVs. At the optimum treatment concentration (1 µg mL^−1^), ApoV uptake was completed after 6 h in all groups (Figure 2F). Live/dead staining showed that the cell survival rate was higher than 95% in all groups within 3 days, indicating that MSC pretreatment does not increase ApoV biotoxicity (Figure 2G,H).

Subsequently, we investigated the effects of each ApoV type on EC function. At the optimum treatment concentration (1 µg mL^−1^), the cell proliferation assay showed that all groups maintained good proliferation, and surprisingly Oxi‐ApoVs group exhibited the highest proliferation, which tentatively suggests that oxidative stress pretreatment has a positive impact on ApoVs (Figure 2I). However, the hypoxia‐protective effect of Con‐ApoVs and Oxi‐ApoVs was observed to be more remarkable in the hypoxia‐injured ECs (Figure S2D, Supporting information). In addition, we assessed the biocompatibility of vascular smooth muscle cells (VSMCs) as mature vessel components. The optimum treatment concentration of ECs had little side effect on the viability of VSMCs (Figure S2E, Supporting information). The scratch migration and in vitro tube formation assay further confirmed the prominent effect of Oxi‐ApoVs at the optimum treatment concentration (1 µg mL^−1^). The migration of ECs, in all experimental groups, significantly increased at 24 h. Con‐ApoVs and Hypo‐ApoVs administration enhanced wound healing from 35% to 53% and 61%, respectively. In addition, the effect was most significant at 68% with the administration of Oxi‐ApoVs (Figure 2J,K). The same trend was observed with tubule formation; Oxi‐ApoVs had the most significant effect on tubule formation. Oxi‐ApoVs administration resulted in the highest number of junctions and meshes as well as the greatest total tubule length. As the most significant representation of the EC function, the number of meshes in Oxi‐ApoVs group counted under 40× magnification visual field was 66, which was 4 and 2.5 times higher than that of Con‐ApoVs and Hypo‐ApoVs groups, respectively (Figure 2L,M). Based on these results, we suggest that alterations are made in the bioactive substances carried by oxygen‐related environmental stressed ApoVs, which may be partially similar or identical because most of the results exhibit a similar trend.

### 2.3 Simultaneously Altered miRNAs in Different Oxygen‐Related Environmental Stressed MSC‐ApoVs Regulate Tissue Regeneration Pathways and EC Signal Transduction

**Figure 3:**
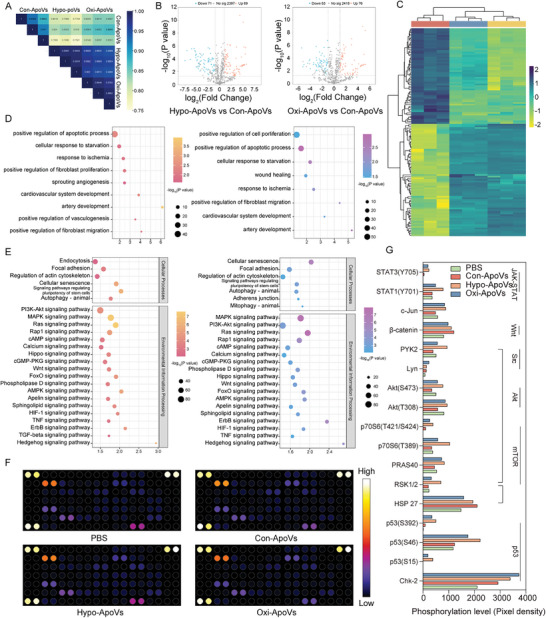
Altered miRNAs in oxygen‐associated environmental stressed ApoVs exhibit a similar trend and upregulate several signaling pathways to activate ECs. A) Heatmap of the Pearson correlation‐based assessment results for the nine ApoVs samples. B) Volcano plots of DEmiR in Hypo‐ApoVs and Oxi‐ApoVs compared to Con‐ApoVs, respectively. C) Heatmap of commonly altered miRNAs in Hypo‐ApoVs and Oxi‐ApoVs compared to Con‐ApoVs. Color depth represents log2 (signal value). D) GO terms related to damage repair and angiogenesis for the target genes of DEmiR. Left: Hypo‐ApoVs versus Con‐ApoVs; right: Oxi‐ApoVs versus Con‐ApoVs. E) KEGG pathway of the target genes, classified as “Cellular Processes” and “Environmental Information Processing” terms (*p* < 0.01). Left: Hypo‐ApoVs versus Con‐ApoVs; right: Oxi‐ApoVs versus Con‐ApoVs. F,G) Representative heatmaps and quantitative analysis of phosphorylated protein arrays.

miRNAs are important communication substances carried by EVs that regulate protein expression in recipient cells and function as post‐transcriptional regulators. Distinct EVs have characteristic miRNA expression profiles that can be distinguished.^[^ ^19^ ^]^ Motivated by cellular functionality experiments, miRNA microarray analysis was used to investigate the co‐altered miRNAs in candidate MSC‐ApoVs from different types of oxygen‐related stress environments. We performed miRNA microarray analysis for Con‐ApoVs, Hypo‐ApoVs, and Oxi‐ApoVs, using three replicates in each group. In total, we identified 2570 miRNAs. The obtained results may reveal the miRNAs responsible for the promotion of angiogenesis. The correlation of all kinds of ApoVs miRNA profiles was analyzed using the Pearson correlation coefficient. As shown in **Figure** **3**A, the miRNA profiles in Hypo‐ApoVs and Oxi‐ApoVs were similar but differed from those of Con‐Abs. Next, we screened for miRNAs that were significantly differentially expressed between the two groups (Hypo‐ApoVs and Oxi‐ApoVs) and Con‐ApoVs group (two‐fold increase or decrease in expression and p‐value<0.05). Compared to Con‐ApoVs (2557 miRNAs), 160 and 139 miRNAs were differentially expressed in Hypo‐ApoVs (89 up‐regulated and 71 down‐regulated) and Oxi‐ApoVs (76 up‐regulated and 63 down‐regulated), respectively (Figure 3B; Figure S3A, Supporting information). Surprisingly, 124 of the differentially expressed miRNAs in the two groups were similarly up‐ or down‐regulated (Figure 3C).

miRNA‐mRNA pair predictions in both miRDB and miRWalk databases were considered to be the target gene relationship pair (Figure S3B, Supporting information). GO and KEGG pathway enrichment analyses were performed to explore the biological functions regulated by these differentially expressed miRNAs(DEmiRs) in oxygen‐related environmental stressed ApoVs. Figure S3C shows the Top ten entries among the GO categories. Biological processes, cellular components, molecular functions, and signal pathways of the DEmiRs target genes were explored. These groups share five out of the ten GO entries in the biological process. The results showed that a large number of DEmiRs target genes in both groups are related to the biological processes of “transcription.” Moreover, the Oxi‐ApoVs miRNAs are involved in the “positive regulation of cell proliferation” and “cell cycle,” whereas the Hypo‐ApoVs miRNAs are involved in “protein transport” and “protein phosphorylation.” The cellular components and molecular functions of the target genes turned out to be similar in both groups, which were mainly labeled in “nucleus” and “DNA binding transcription factor activity.” In addition, entries like “intracellular signal transduction,” “protein phosphorylation,” and “protein kinase binding” suggest that the miRNAs are involved in the regulation of tissue regeneration. Considering that ApoVs promotes angiogenesis in oxygen‐stressed environments, we analyzed the GO terms related to damage repair and angiogenesis (Figure 3D). While the same entries from the two groups focus on cellular responses to abnormal environments and vasculature development; DEmiRs in Hypo‐ApoVs are involved in “sprouting angiogenesis” and “positive regulation of vasculogenesis,” while those in Oxi‐ApoVs are more broadly implicated in “wound healing” and “positive regulation of cell proliferation.” KEGG analysis showed that these DEmiRs significantly participate in the “PI3K‐Akt signal pathway,” “MAPK signal pathway,” “Wnt signal pathway,” and “HIF‐1 signal pathway” etc., which are important pathways in tissue regeneration and angiogenesis. In addition, “autophagy,” “cellular senescence,” and “stem cell pluripotency” were also highlighted, hinting at the great potential of ApoVs in the field of autophagy linked to disease and aging (Figure 3E).

To determine how signal pathways in ECs are affected by Con‐ApoVs and oxygen‐stressed ApoVs, we performed phosphokinase antibody array analyses of ECs treated with different ApoVs (Figure 3F; Figure S3D, Supporting information). Of the 43 evaluated kinases, the levels of 12 increased in at least one group. Among these, the enhanced activation of several kinases related to Chemokine/Akt/mTOR signal pathway was confirmed. In addition, kinases associated with Wnt and JAK‐STAT pathways related to EC viability and angiogenesis were up‐regulated (Figure 3G). Surprisingly the level of p53 and its upstream signal, which regulates cell cycle and cell death, was elevated. HSP27 is known as an antioxidant and anti‐apoptotic protein and is involved in actin‐associated cytoskeletal remodeling in Ecs.^[^ ^20^ ^]^ The RSK2‐induced increase in HSP27 phosphorylation may be an endogenous inhibition of the p53 apoptotic pathway.^[^ ^21^ ^]^ This could partly explain the limited therapeutic window of MSC‐ApoVs (Figure S2A,B, Supporting information).

Combining the ApoV miRNA microarray and EC phosphokinase antibody array results, it was found that there are similarities in the miRNA profiles of oxygen‐environmentally stressed ApoVs compared to ApoVs in a normoxic environment. These similar miRNAs regulate transcriptional processes in ECs. This may be the result of the intricate intracellular crosstalk between hypoxia and oxidative stress. For example, NADPH oxidase activation requires HIF‐1α accumulation; conversely, HIFs are induced by ROS stimulation.^[^ ^22^ ^]^ We predicted a number of altered biological functions and molecular pathways by GO and KEGG analyses, some of which were validated by phosphokinase antibody array analysis. Most of the mentioned biological processes were related to positive regulation of cell growth, transcription, translation, and cell cycle and function. KEGG analysis showed that the PI3K‐Akt signaling axis plays an important role in such processes, which was also confirmed in EC phosphokinase assays. PI3K‐Akt signaling axis is activated by a variety of stimuli in ECs and regulates multiple critical steps in angiogenesis, including EC survival, migration, and capillary‐like structure formation. Furthermore, it also regulates cardiovascular homeostasis and NO synthesis.^[^ ^23^ ^]^ Besides, other molecular pathways include Wnt, MAPK, HIF‐1, Ras pathway, and so on. These potential signaling mechanisms need to be further explored. In addition, clues about autophagy and apoptosis have also attracted our attention. The AMPK, Hippo, and FoxO pathways are all closely related to autophagy. The characteristic difference between ApoVs and other EVs derived from the paracrine pathway is that the cargoes contain DNA fragments derived from apoptotic parent cells,^[^ ^6^ ^]^ which may explain the accumulation of the p53 protein and apoptosis of the recipient cells.^[^ ^24^ ^]^ However, the activation of autophagy and p53 pathway and safety evaluation still need to be further explored. In conclusion, DEmiRs in ApoVs subjected to oxygen‐related environmental stress are involved in a series of molecular pathways related to angiogenesis represented by PI3K‐Akt.

### 2.4 ApoVs Regulate EC Function Through the Akt Pathway, and Oxygen‐Related Environmental Stressed ApoVs Promote EC Migration via miR‐210‐3p‐Induced Akt Signal Activation

**Figure 4:**
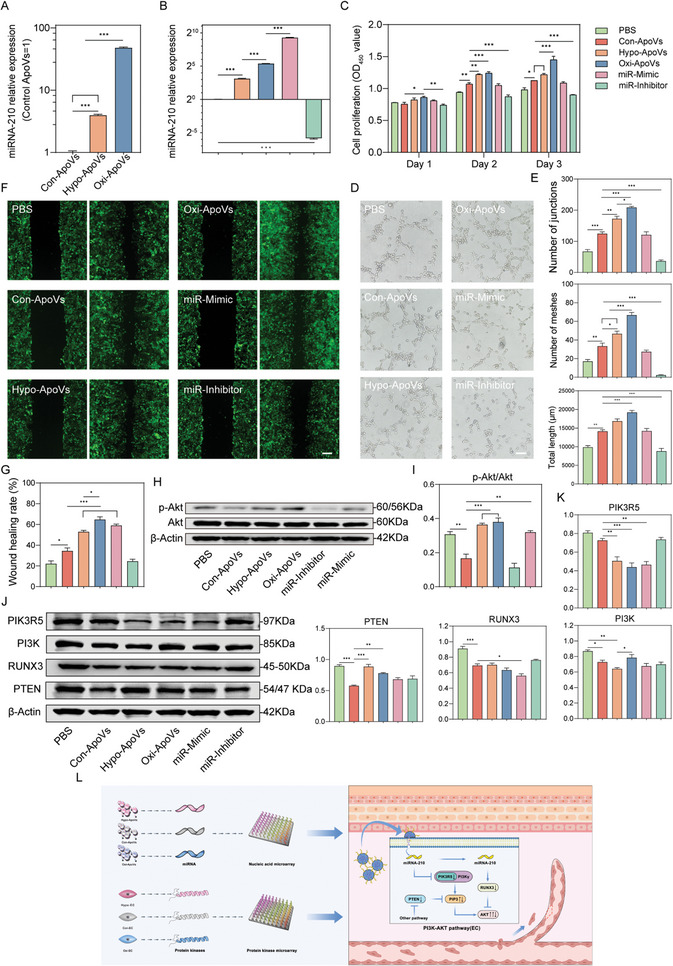
The highly expressed miR‐210‐3p in oxygen‐related environmental stressed ApoVs is responsible for ECs migratory improvement through Akt pathway. A) qPCR analysis of miRNA‐210‐3p expression in Con‐ApoVs, Hypo‐ApoVs and Oxi‐ApoVs. B) qPCR analysis of miRNA‐210‐3p expression after transfection with miRNA‐210‐3p mimics and inhibitors. C) Effect of miRNA‐210‐3p mimics or inhibitors on EC proliferation. D–G) Qualitative and quantitative analyses of the scratch and the tube‐formation assay. Scale bar  =  100 µm. H,I) Expression and quantitative analysis of phosphorylated Akt and total Akt proteins in ECs treated with Con‐ApoVs, Hypo‐ApoVs, Oxi‐ApoVs, miR‐Inhibitor, or miR‐Mimic. J,K) Expression and quantitative analysis of PIK3R5, PI3K, RUNX3, and PTEN proteins in ECs treated with ApoVs in each group. L) Mechanisms of miRNA‐210 promoting endothelial cell migration. ^*^ *p* < 0.05, ^**^ *p* < 0.01, ^***^ *p* < 0.001 (*n* = 3, biological replicates for each group).

To investigate how ApoVs derived from oxygen‐stressed environments act on the Akt signal pathway to exert specific effects on ECs, we considered the overlapping portion of the top 20 miRNAs up‐regulated in Hypo‐ApoVs and Oxi‐ApoVs groups and correlated them with the miRNA predictions under the PI3K‐Akt signal pathway entries in the KEGG enrichment analysis. MiR‐210‐3p and miR −208‐5p were ultimately identified. In addition, miR‐424‐3p, which has been reported to be a regulator of cell growth and apoptosis among the up‐regulated miRNAs, was also detected.^[^ ^25^ ^]^ Among these miRNAs, changes in miR‐210‐3p levels were consistent with the trend of EC activation (**Figure** **4**A; Figure S4A,B, Supporting information), and miR‐210 has also been shown to enhance angiogenesis.^[^ ^26^ ^]^

Next, we constructed MSC‐ApoVs transfected with a miR‐210‐3p mimic or inhibitor (miR‐Mimic and miR‐Inhibitor) (Figure 4B). Surprisingly, the cell proliferation assay showed that miR‐210‐3p mimic did not elicit EC proliferation‐enhancing properties; however, miR‐210‐3p inhibitor eliminated the positive effects of ApoVs (Figure 4C). Tube formation experiments yielded similar results (Figure 4D,E). However, scratch experiments showed distinct results. The miR‐210‐3p inhibitor eliminated the effects of Con‐ApoVs while the miR‐Mimic group showed satisfactory migration enhancement of 58.9% compared to 52.8% in Hypo‐ApoVs group (Figure 4F,G). Western blotting results were also surprising. As shown in Figure 4H,I, the Akt phosphorylation levels were up‐regulated in Hypo‐ApoVs, Oxi‐ApoVs, and miR‐Mimic groups and down‐regulated in miR‐Inhibitor group, which demonstrates that miR‐210‐3p is a specific agonist of the Akt pathway in oxygen‐related environmental stressed ApoVs. Compared to that in untreated ECs, the low Akt phosphorylation level in Con‐ApoVs group suggests the existence of other pathways mediating endothelial activation in MSC‐ApoVs, such as the potential induction of autophagy.^[^ ^27^ ^]^ We also verified that miR‐210‐3p represents a specific STAT3 agonist (Figure S4C, Supporting information). STAT3 is another perceived activated site in the phosphokinase microarray. Overall, in this study, the high level of miR‐210‐3p in oxygen‐stressed ApoVs induces endothelial migration, rather than angiogenesis, through the activation of Akt pathway. These results concur with those of Liu et al.^[^ ^28^ ^]^ Furthermore, although Akt is not the primary pathway for MSC‐ApoVs to improve EC function, its inactivation actually reverses the positive effects of MSC‐ApoVs. Thus, activation of the Akt pathway is essential for the regulation of EC function by MSC‐ApoVs.

Finally, we sought to explore the mechanisms by which miR‐210‐3p directs the Akt pathway. The target genes of miR‐210‐3p were predicted using a combination of miRWalk 3.0 and miRDB databases. PIK3R5, RUNX3, and PTEN were selected due to their relevance to the core processes in the PI3K‐Akt pathway. PIK3R5 is a regulatory subunit of PI3Kγ in class Ib PI3K, which activates Akt by catalyzing the phosphorylation from PIP2 to PIP3. PTEN, in turn, acts as a phosphatase to antagonize this process. RUNX3 is a transcriptional inhibitor of Akt1 and could reduce the level of Akt phosphorylation. Figure 4J,K shows the western blotting results for these proteins. PIK3R5 and RUNX3 were negatively regulated by miR‐210‐3p. PI3K expression levels were partially changed by PIK3R5 level. There are other unidentified factors that could be responsible for PI3K up‐regulation in the Oxi‐ApoVs group, which may explain the higher Akt phosphorylation levels. It is well known that miRNAs have quite complex mechanisms of action. A single miRNA can regulate multiple mRNA targets and vice versa.^[^ ^29^ ^]^ PIK3R5 and RUNX3 cannot fully account for the changes in Akt pathway, and other direct targets have been reported, such as PTP1B, DAPK1^[^ ^30^ ^],^ and PDK1.^[^ ^31^ ^]^ The prediction of PTEN proved to be wrong, but the distinct expression trends caused by oxygen‐stressed ApoVs and Con‐ApoVs require further exploration. Our results suggest that miR‐210‐3p is a major, but not the only, mediator of oxygen‐related environmental stressed ApoVs promoting EC migration through up‐regulation of the Akt pathway (Figure 4L).

### 2.5 Hydrogel Microspheres Control Oxi‐ApoVs Release

As shown in Figure 1, systemically injected MSCs and ApoVs had similar positive effects on wound healing; however, differences in the oxygen‐related microenvironment in vivo and the action time led to differences in outcome. Oxi‐ApoVs demonstrated a more pronounced pro‐angiogenic effect in vitro experiments (Figure 2) and were therefore chosen for in vivo experiments. In wound healing, Days 3 to 14 are the proliferative phase, as well as the main period of angiogenesis, marked by fibroblast migration, extracellular matrix deposition, and granulation tissue formation, while Day 1 to 2 is the inflammatory phase.^[^ ^10^ ^]^ We suspected that the delayed release brought about by apoptosis of MSC transplantation in vivo promotes EC uptake, as no specific uptake signals for inflammatory or endothelial cells have been found on MSC‐ApoVs.^[^ ^32^ ^]^ The GelMA‐sodium alginate microspheres (*GA‐MSP*) were fabricated in the hope of simulating the natural controlled release in cell therapy.

**Figure 5:**
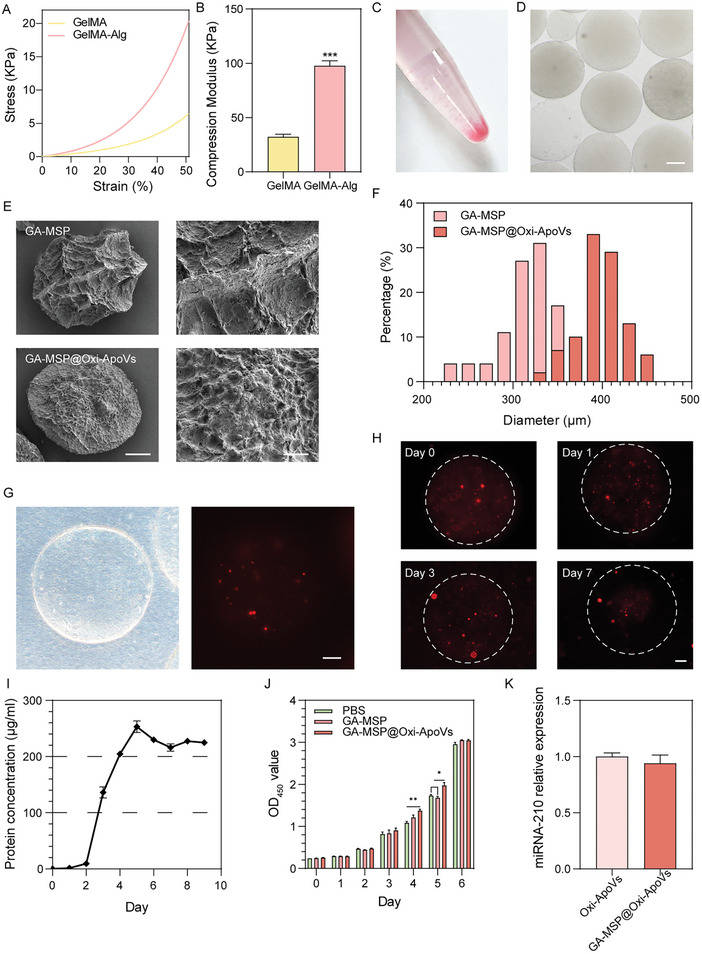
Characterization of GelMA‐sodium alginate hydrogel microspheres loaded with Oxi‐ApoVs (GA‐MSP@Oxi‐ApoVs). A) Stress‐strain curves of hydrogels. B) Elastic modulus of hydrogels. C) *GA‐MSP* gross morphology. D) Optical images of GA‐MSP. Scale bar  =  100 µm. E) SEM images of freeze‐dried microspheres. Left: scale bar  =  50 µm, right: scale bar  =  10 µm. F) Particle size distributions of GA‐MSP and GA‐MSP@Oxi‐ApoVs. G) Optical and immunofluorescence images of a microsphere loaded with PKH26‐labeled Oxi‐ApoVs. Scale bar  =  50 µm. H)Fluorescence images of Oxi‐ApoVs released from microspheres at different time points. Scale bar  =  50 µm. I) Oxi‐ApoVs release curves. J) Proliferation of ECs co‐cultured with GA‐MSP and GA‐MSP@Oxi‐ApoVs. K) qPCR analysis of miRNA‐210‐3p in Oxi‐ApoVs and GA‐MSP@Oxi‐ApoVs. ^*^ *p* < 0.05, ^**^ *p* < 0.01, ^***^ *p* < 0.001 (*n* = 3, biological replicates per group).

GelMA is a photo‐crosslinked hydrogel synthesized by replacing the lysine and hydroxylysine of gelatin with methacrylic anhydride (MA).^[^ ^33^ ^]^ GelMA presents natural cell‐binding motifs such as RGD and MMP‐sensitive degradation sites. The modified MA occupies less than 5% of the amino acid residues.^[^ ^34^ ^]^ The ^1^H NMR spectral results of GelMA are shown in Figure S5A (Supporting Information). The new signals at 5.4 and 5.6 ppm indicate acrylic protons of methacrylamide groups, suggesting that the MA modification was successful. The degree of methacrylation was determined to be 40.63%. Due to the thermal instability of gelatin, when the temperature is below 25 °C, the shear modulus increases significantly.^[^ ^35^ ^]^ However, extracellular vesicles are difficult to maintain activity at high temperatures.^[^ ^36^ ^]^ And the Young's modulus of GelMA is relatively low.^[^ ^34^ ^]^ Therefore, the combination of interpenetrating polymeric networks of GelMA and sodium alginate is used to encapsulate cells with excellent biocompatibility, low immunogenicity, appropriate stiffness, and a mild cross‐linking environment.^[^ ^37^ ^]^ The 4% GelMA solution gels at low temperatures, whereas the GelMA and sodium alginate mixture does not (Figure S5B, Supporting information). As the elastic modulus of microspheres closely matches that of the bulk hydrogels calculated from uniaxial compression,^[^ ^38^ ^]^ we measured the elastic modulus of GelMA and GelMA‐Alg hydrogels(**Figure** **5**A,B). The modulus of GelMA‐Alg hydrogel is more similar to that of skin tissue.^[^ ^39^ ^]^ In addition, alginate hydrogel is crosslinked at room temperature which maintains the morphology of the microspheres at an early stage, so that GelMA photocrosslinking takes place at room temperature, thus reducing ApoVs lost through the freeze‐thaw cycle.^[^ ^40^ ^]^

GelMA‐alginate microspheres (*GA‐MSP*) or microspheres with Oxi‐ApoV preloaded were fabricated using a co‐axial microfluidic device (Figure S5C, Supporting information). The gross and microscopic images of the microspheres are shown in Figure 5C,D. Scanning electron microscopy (SEM) showed Oxi‐ApoV attachment on *GA‐MSP@Oxi‐ApoVs* (Figure 5E). The diameters of *GA‐MSP* and *GA‐MSP@Oxi‐ApoVs* were 313.58 ± 29.93 and 397.76 ± 24.96 µm, respectively, allowing for injection through a 1 mL syringe needle (Figure 5F). Fluorescence staining confirmed that Oxi‐ApoVs in *GA‐MSP@Oxi‐ApoVs* were uniformly distributed (Figure 5G, Movie S1, Supporting information). To assess the therapeutic potential of the injectable microspheres, we investigated the release capacity of *GA‐MSP@Oxi‐ApoVs*. As shown in Figure 5H, ApoVs were released in large quantities from Day 3 to 5, which is tentatively consistent with the temporal clues of MSC apoptosis in situ. The fluorescence intensity of the red‐labeled ApoVs was proportional to the protein concentration (Figure S5D, Supporting information), from which we investigated the release profile of ApoVs. As shown in Figure S5E and Figure 5I, there was limited ApoV release for the first two days, with a clear burst from Day 3 to Day 5. The release pattern closely fits the time window of neovascularisation for tissue healing and resembles the in situ apoptotic secretion pattern of transplanted MSCs. Besides, we demonstrated that *GA‐MSP@Oxi‐ApoVs* significantly enhanced the proliferation of ECs on days 4 and 5 (Figure 5J) by the CCK‐8 assay. In addition, qPCR verified that hydrogel crosslinking did not reduce the transport efficiency of miRNA. No significant difference was observed before and after miR‐210‐3p microsphere loading (Figure 5K). The results suggest that *GA‐MSP* is an effective controlled‐release delivery vehicle for Apovs that exerts MSC‐like secretion.

### 2.6 Local Injection of GA‐MSP@Oxi‐ApoVs Effectively Promotes Chronic Wound Healing and Angiogenesis

**Figure 6:**
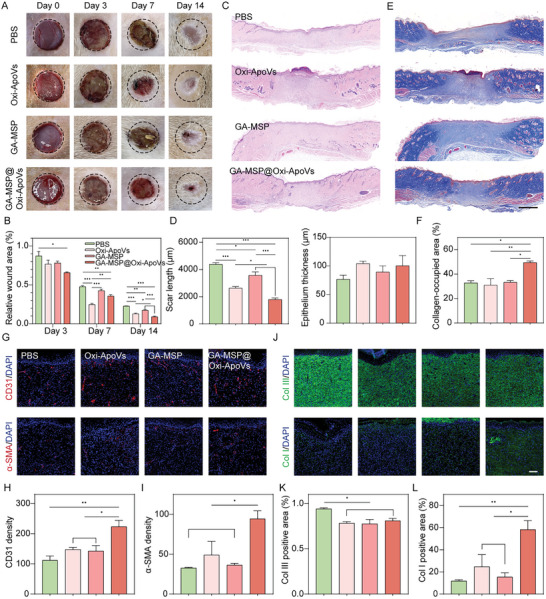
Local injection of GA‐MSP@Oxi‐ApoVs promotes wound healing in diabetic rats by improving angiogenesis and collagen deposition. A,B) Representative images of wounds treated with Oxi‐ApoVs, GA‐MSP, or GA‐MSP@Oxi‐ApoVs at different time points and wound closure area measurement. C) Typical images of HE staining on day 14. D) Quantitative analysis of the wound length and epidermal thickness. E) Typical images of Masson trichrome staining. Scale bar  =  1000 µm. F) collagen deposition rate. G,J) Typical images of CD31, α‐SMA, COL‐III, and COL‐I fluorescence staining. Scale bar  =  100 µm. H,I) CD31 and α‐SMA fluorescence density. (K‐L) COL‐III, and COL‐I fluorescence density. ^*^ *p* < 0.05, ^**^ *p* < 0.01, ^***^ *p* < 0.001 (*n* = 3, biological replicates per group).

A rat diabetic wound model was established to demonstrate the efficacy of local *GA‐MSP@Oxi‐ApoVs* injection in tissue injury. Microspheres or Oxi‐ApoVs were injected subcutaneously around the wound during surgery. The results showed that all injections significantly increased wound closure in diabetic rats on Day 14 (**Figure** **6**A). In particular, Oxi‐ApoVs group had a higher wound healing rate than the other groups on day 7, but *GA‐MSP@Oxi‐ApoVs* had the best healing effect on day 14 (Figure 6B). This again suggests that the delayed release of ApoVs results in better outcomes. Histological assessments, including HE staining and Masson trichrome staining, were performed on Days 7 and 14 to quantify the degree of re‐epithelialization, scar formation, and collagen regeneration. We found that the wound sites had been filled with granulation tissue in all experimental groups on day 7. Re‐epithelialization was observed only at the wound edges in all groups. Oxi‐ApoVs group had the shortest scar length (Figure S6A,B, Supporting information). Masson staining showed that all experimental groups exhibited collagen deposition; Oxi‐ApoVs group exhibited a significantly higher collagen content and a local orderly collagen fiber arrangement (Figure S6C,D, Supporting information). However, *GA‐MSP@Oxi‐ApoVs* group was set to surpass on day 14. HE staining showed that the shortest wound length was observed in *GA‐MSP@Oxi‐ApoVs*‐group, and there was also a significant difference between Oxi‐ApoVs group and the other two groups. Skin appendages could be observed in both oxygen‐stressed groups. However, there was no significant difference in the level of re‐epithelialization (Figure 6C,D). Masson staining showed the highest collagen density in *GA‐MSP@Oxi‐ApoVs* group, with a reduction of vesicle‐like structures, and an increased arrangement of mature bundles of collagen fiber (Figure 6E,F). In conclusion, Oxi‐ApoVs promote early and rapid wound healing and the controlled release of *GA‐MSP@Oxi‐ApoVs* improves wound healing outcomes in diabetic rats.

Angiogenesis and collagen deposition are the main activities during the proliferative phase of tissue healing. To assess the effect of different treatments on the vascularization of diabetic wounds, the endothelial marker CD31 and the smooth muscle cell marker α‐SMA were evaluated by immunofluorescence staining. The neovascularization results were similar to morphological and Histopathology findings. The Oxi‐ApoVs group had the highest number of CD31^+^ neovessels on day 7, but the *GA‐MSP@Oxi‐ApoVs* group showed a better outcome on day 14. Surprisingly the number of α‐SMA^+^ vessels in the *GA‐MSP@Oxi‐ApoVs* group significantly exceeded others on both day 7 and day 14 (Figure 6G–I; Figure S6E–G, Supporting information). α‐SMA is a sign of blood vessel maturation, suggesting that the neovessels were wrapped by smooth muscle cells. In addition, the content and proportion of collagen types I and III were also investigated. On day 7, the PBS and *GA‐MSP@Oxi‐ApoVs* groups showed better Col III deposition (Figure S6E,H, Supporting information). On day 14, the percentage of Col III decreased in the *GA‐MSP@Oxi‐ApoVs* group, while it remained at a high level in the PBS group (Figure 6J,K). Correspondingly, there was more Col I deposition in the *GA‐MSP@Oxi‐ApoVs* group compared to the other groups (Figure 6J,L). The high level of Col III is characteristic of the early stages of wound healing. As the scar matures, Col I/Col III ratio goes up and becomes close to that of normal tissue. As shown in Figure S6I (Supporting Information), Col I/Col III ratio was highest in *GA‐MSP@Oxi‐ApoVs* group. In addition, Col I is thought to stimulate angiogenesis.^[^ ^41^ ^]^ Finally, we verified the oxygen‐abnormal microenvironment again in the wound and found that a continuous accumulation of ROS in the wound area was still detectable on day 7 (Figure S6E, Supporting information). In conclusion, in vivo, experiments demonstrated that the controlled release of Oxi‐ApoVs via *GA‐MSP@Oxi‐ApoVs* optimally promoted neovasculogenesis as well as early vascular and collagen maturation.

The clinical implementation potential of ApoVs has attracted much attention as a promising cellular substitutive therapy.^[^ ^42^ ^]^ Currently, ApoV is still under‐explored in preclinical and clinical studies. Among the cells that play an important role in tissue healing, inflammatory cells, and endothelial cells have been shown to be regulated by ApoV. However, the role of ApoVs in other related cells, such as vascular smooth muscle cells and fibroblasts, remains unknown. Although our findings suggest that Oxi‐ApoV may have therapeutic effects on these cells, further studies are necessary to validate these effects and identify the mechanisms involved. Similar to existing therapies that promote tissue repair, a single intervention that can repair nonhealing tissue defects effectively is simply impossible, as it is widely recognized as a complex process.^[^ ^43^ ^]^ We have altered the miRNA delivery of Oxi‐ApoV by manipulating the oxygen culture environment of the parental MSC. In the future, it would be interesting to explore the synergistic benefits of Oxi‐ApoV with other therapies, which may further amplify the pro‐repair efficacy of Oxi‐ApoV. In addition, challenges for clinical applications would include the incorporation of more precise membrane‐specific molecules or stimuli‐triggered modification in the biomaterials to improve recipient cell targeting^[^ ^44^ ^]^ and the enhancement of ApoV stability for preservation,^[^ ^17^ ^]^ which will greatly increase the clinical utility of ApoVs.

## 3 Conclusion

In summary, we show that oxygen‐stressed ApoVs exert superior pro‐angiogenic efficacy through a series of pathways, in which miR‐210‐3p is responsible for the migration of ECs by upregulating the repressed Akt expression. The local delivery and controlled release of hydrogel microspheres loaded with Oxi‐ApoVs was designed to mimic the release of ApoVs from apoptotic MSCs in vivo. It is aimed to increase the proportion of EC uptake and maintain the in vitro activity of ApoVs. Additionally, we have shown that Oxi‐ApoVs improve Col I/Col III deposition, which highlights the potential efficacy of apoptotic vesicles on other recipient cell populations in the repair process. In conclusion, the current findings provide promising avenues for the development of ApoV‐based tissue regeneration therapies.

## 4 Experimental Section

### Biogenesis and Characterization of ApoVs

First, MSCs (Anwei Biotechnology, Shanghai, China) were incubated at 37 °C and 5% CO_2._ CoCl_2_·6H_2_O (100 µM) (Sigma‐Aldrich, St. Louis, MO) or H_2_O_2_(50 µM) (Macklin, Shanghai, China) was diluted in DMEM and used immediately. The cell culture medium was replaced after 24 h.^[^ ^15^, ^16^ ^]^ After that, in accordance with the previously published research methodology and other similar studies, the cells were further incubated for 12 h in serum‐free DMEM with 5 µM staurosporine (STS, 5 µM) (Sigma‐Aldrich, St. Louis, MO) to induce the apoptotic process. Apoptosis progression was observed using light microscopy. After trypsin digestion, cell products were collected from a petri dish and centrifuged at 300 g for 10 min to remove large debris, and the supernatant was carefully collected. Then, a subsequent centrifugation step was performed at 3000 g for 10 min to obtain ApoVs of the appropriate size, named Hypo‐ApoVs, Oxi‐ApoVs, or Con‐ApoVs.^[^ ^12^, ^45^ ^]^ ApoVs were washed twice with PBS and resuspended and stored at −80 °C for subsequent experiments. Protein concentrations of ApoVs were determined using the BCA protein assay kit (abs9232, Absin, Shanghai, China) following the manufacturer's guidelines. ApoVs were stained by C1q (abs04012, Absin, Shanghai, China) and the Membrane Linker V‐FITC/PI Apoptosis Kit (abs50001, Absin, Shanghai, China). Visualization was achieved through an inverted fluorescence microscopy (Eclipse Ts2, Nikon, Tokyo, Japan) and the markers were analyzed by a flow cytometer (BD, Accuri C6 Cytometer, USA). The morphology of ApoVs was examined by transmission electron microscope. On a carbon‐coated copper grid, 20 µL of ApoVs suspension was simply loaded and set for at least 5 min. The ApoVs was subsequently stained with 2% phosphotungstic acid and dried for 1 min. Then, the grids were visualized with a transmission electron microscope (HT‐7700, Hitachi, Japan) at 120 kV. DLS diameters and zeta potential were measured by Zetasizer Nano ZSE (Malvern, Malvern, UK).

### ECs Functional Experiments

ApoVs were labeled with PKH26 (MINI26, Sigma‐Aldrich, St. Louis, MO) following the manufacturer's instructions. The labeled ApoVs were then co‐cultured with ECs in DMEM complete medium for 6 h. The cells were fixed with 4% paraformaldehyde, and the cytoskeleton and nuclei were stained using FITC ghost‐pen cyclic peptide and 4′,6‐diamidino‐2‐phenylindole (DAPI) (P5282, D9542, Sigma‐Aldrich, St. Louis, MO) and phagocytosis was observed by confocal microscope (Leica STELLARIS 5, Wetzair, Germany). For scratch assays, 1 × 10^6^ ECs were inoculated in six‐well plates and incubated at 37 °C. The monolayer was scratched with a p200 pipette tip after the cells had been attached, and the plate was washed with PBS to remove floating cells. ECs were photographed after 24 h. Mobility was calculated by analyzing the healing area of scratches by ImageJ software. The following formula was used to calculate the percentage migration area. Migration area (%) = (A1 – A0)/A0 × 100, where A1 represents the initial wound area and A0 represents the remaining wound area at the metering point. EC angiogenic capacity was determined in Matrigel by performing a tube formation assay. The 1 × 10^5^ ECs were seeded on Matrigel (BD, New Jersey, USA) coated 24‐well confocal plates with 100 uL of serum‐free medium, and tube formation was assessed by microscopy after 6 h of incubation. Three random 40× images of each well were taken with a light microscope. Quantitative analysis was conducted by measuring the number of branching points and meshes, as well as the tube lengths using Angiogenesis Analyzer for ImageJ (National Institutes of Health, Bethesda, Maryland).

### Transfection of Mimics and Inhibitors

The miR‐210‐3p mimic (50 nmol L^−1^) or inhibitor (100 nmol L^−1^), or its corresponding negative control (GenePharma, Shanghai, China) was transfected into MSCs (≈6 × 10^5^ cells/dish) in 10‐cm petri dishes. GP‐transfect‐Mate (GenePharma, Shanghai, China) transfection reagent was also added to the medium for efficient transfection. After 6 h, the medium was replaced to remove the transfection reagent.

### Fabrication of Microspheres

A 30G needle was inserted into a 27G needle, and the connection was sealed with resin glue. The 30G/27G co‐axial microfluidic device was used as the microfluidic channel. To facilitate the collection of microspheres, the outlet of the needle was connected to a polyvinyl chloride tube. Lyophilized GelMA (200 mg) and sodium alginate (60 mg) were completely dissolved in 5 mL of PBS along with photoinitiator (20 mg) as the aqueous phase. For ApoVs‐loaded microspheres (GA‐MSP@Oxi‐ApoVs), an additional 250 µg mL^−1^ of ApoVs was included. The continuous oil phase, consisting of paraffin oil containing 5% (w/w) Span 80 (Sigma‐Aldrich, St. Louis, MO), was utilized to stabilize the microsphere droplets formed by shear stress. The flow rate of the water phase and oil phase was adjusted to obtain droplets of optimal sizes.^[^ ^46^ ^]^ The microspheres were cross‐linked by first immersing them in a 1% (w/w) CaCl2 solution, followed by a second cross‐linking under 365 nm blue light irradiation for 30 seconds. Afterward the microspheres were subjected to three rounds of centrifugation at 300 g for 5 min using either PBS or DMEM medium supplemented with 100 µg mL^−1^ streptomycin and 100 U mL^−1^ penicillin to obtain clean microspheres.

### Animal Models and Treatment

Female Balb/c nude mice and Sprague Dawley (SD) rats were obtained for the study. All animal procedures were approved by the Animal Research Committee of Shanghai Jiao Tong University (HKDL‐2018‐141). All in vivo experimental protocols were performed in accordance with institutional animal care guidelines. For Balb/c nude mice, anesthesia and sterilization were administered before three full skin wounds (5 mm diameter) were created on the backs of the mice. PBS (100 µL), MSC suspension (2 × 10^5^ cells), or ApoVs suspension (ApoVs generated from 2 × 10^5^ cells) was infused via the tail vein. Photographs of the wounds at different time points were obtained using a digital camera. Mice were euthanized on days 1, 3, or 7, and wound tissue samples were obtained.

SD rats were injected at body weights up to 180–220 g using streptozotocin (STZ) (50 mg k^−1^g, intraperitoneal) for rapid induction of a type 1 diabetes model. After one week, all blood glucose levels reached 16.7 mmol L^−1^. An 8‐h fast was imposed prior to experimental procedures. Rats were anesthetized by intraperitoneal injection of sodium pentobarbital and their backs were shaved with an electric razor and depilatory cream. Three full‐skin wounds (10 mm in diameter) were created on their backs. The rats were randomly divided into four groups (PBS group, Oxi‐ApoVs group, GA‐MSP group, and GA‐MSP@Oxi‐ApoVs group), and PBS/Oxi‐ApoVs/GA‐MSP/GA‐MSP@Oxi‐ApoVs were injected subcutaneously around the wounds at four sites (25 µL at each site.) Photographs of the wounds at different time points were taken using a digital camera. Rats were euthanized to obtain wound tissue samples on days 7 or 14.

### Statistical Analysis

Statistical analyses were performed using GraphPad Prism software 8 (GraphPad Software, Inc.). All data are shown as mean ± standard error (SEM). The statistical significance of differences was assessed by two‐sided Student's *t*‐test (for comparisons between two groups) or one‐way analysis of variance (ANOVA) with Tukey's test (for comparisons between more than two groups). *p* < 0.05 was considered statistically significant.

## Conflict of Interest

The authors declare no conflict of interest.

## Supporting information

Supporting Information

Supplemental Movie 1

## Data Availability

The data that support the findings of this study are available from the corresponding author upon reasonable request.
