# Supplementary material for: Biogenerated Oxygen‐Related Environmental Stressed Apoptotic Vesicle Targets Endothelial Cells
Source: Adv Sci (Weinh). 2024 Mar 13;11(20):2306555. doi: 10.1002/advs.202306555 (PMC11132028; doi:10.1002/advs.202306555)
Supplement: Supplementary file 1 — Supporting Information [file ADVS-11-2306555-s001.pdf]

ADVANCED SCIENCE
Open Access

Supporting Information

for *Adv. Sci.*, DOI 10.1002/advs.202306555

Biogenerated Oxygen-Related Environmental Stressed Apoptotic Vesicle Targets Endothelial Cells

Qiuyu Zhao, Bolun Lu, Shutong Qian, Jiayi Mao, Liucheng Zhang, Yuguang Zhang, Xiyuan Mao*, Wenguo Cui* and Xiaoming Sun**

Supporting Information

Biogenerated Oxygen-related Environmental Stressed Apoptotic Vesicle Targets Endothelial Cells

Qiuyu Zhao[1], Bolun Lu [1], Shutong Qian, Jiayi Mao, Liucheng Zhang, Yuguang Zhang, Xiyuan Mao*, Wenguo Cui*, Xiaoming Sun**

Dr. Q. Zhao, Dr. B. Lu, Dr. J. Mao, Dr. L. Zhang, Dr. X. Mao, Prof. Y. Zhang, Dr. X. Sun

Department of Plastic and Reconstructive Surgery, Shanghai Ninth People's Hospital, Shanghai JiaoTong University School of Medicine, 639 Zhi Zao Ju Road, Shanghai 200011, P. R. China.

E-mail: drsunxm@126.com (X.Sun.); zhangyg18@126.com (Y.Zhang.); sakimao@126.com (X.Mao.).

Dr. S. Qian

Department of Plastic Surgery, The First Affiliated Hospital, College of Medicine, Zhejiang University, Hangzhou 310003, China

Prof. W. Cui

Department of Orthopaedics, Shanghai Key Laboratory for Prevention and Treatment of Bone and Joint Diseases, Shanghai Institute of Traumatology and Orthopaedics, Ruijin Hospital, Shanghai Jiao Tong University School of Medicine, 197 Ruijin 2nd Road, Shanghai 200025, P. R. China.

E-mail: wgcui80@hotmail.com.

Keywords: oxygen-related stress; apoptotic vesicles; angiogenesis; microspheres;

Supplementary Materials and Methods

Cell biocompatibility test: The ApoVs were co-cultured with endothelial cells (HUVECs, FDCC, China) or vascular smooth muscle cells (HA-VSMCs, FDCC, China) in DMEM with 10% FBS. Cell viability was assessed using the CCK-8 kit (CK04, Dojindo, Kumamoto, Japan). Hypoxic endothelial cells were incubated with 100 μmol/L cobalt chloride for 24 h. Optical density (OD) values of the various groups were measured at 450 nm using an enzyme marker (Varioskan Flash 3001, Thermo Fisher Scientific, Massachusetts, USA). On day 1, 2 and 3, cells were stained using a live/dead cell double staining kit (Sigma-Aldrich, St. Louis, Missouri, USA) for 30 min at room temperature and then examined under a invert fluorescence microscope. Live and dead cell quantifications were performed using ImageJ software and cell viability was calculated as the ratio of the number of live cells to the total number of cells. The biocompatibility of different concentrations and types of ApoVs with ECs was assessed in a similar way.

Microarray analysis of miRNAs: The miRNA microarray assay (Agilent Human miRNA Microarray Kit, Release 21.0,8x60K, DesignID:070156; Agilent Technologies Deutschland GmbH, Waldbronn, Germany) and data analysis of the nine ApoVs samples were performed by OE Biotechnology Co. (Shanghai, China). Feature Extraction software (version 10.7.1.1; Agilent Technologies Deutschland GmbH, Waldbronn, Germany) was used to analyze the array images to obtain raw data. Raw data were normalized using quantification algorithms. Differently expressed ApoV miRNAs were subsequently identified based on their fold change as well as p value. Thresholds for up- and down-regulated genes were set at $FC \geq 2.0$ and $p \leq 0.05$. Target genes were predicted based on miRWalk 3.0 and miRDB database. Based on the hypergeometric distribution, the screened miRNA target genes were analyzed for GO and KEGG pathway enrichment.

Quantitative real-time polymerase chain reaction (qPCR): TRIzol was used to extract

total RNA. The concentration and purity of RNA determined through optical densitometry at 260 and 280 nm. Reverse transcription was obtained using the TaqManmiR Reverse Transcription Kit (Applied Biosystems, Foster City, CA, USA) according to the manufacturer's instructions. U6 (U6 small nucleolar RNA [snRNA]; assay ID: 001973) was selected as the housekeeping gene. Real-time PCR amplification was performed in triplicate. quantStudio 6 Flex Real-Time PCR system (Applied Biosystems, Foster City, CA, USA) was used to quantify cDNA fragments for PCR. Real-time PCR amplification was carried out in triplicate. Relative mRNA expression was assessed using the 2-ΔΔCt method

Western blotting and phospho-kinase array: Samples were lysed with a protein extraction kit supplemented with protease and phosphatase inhibitors (#78501, #89842, Thermo Fisher Scientific, Waltham, MA, USA). After quantification with BCA kit, 20 μg of protein from each sample was supersampled onto SDS-PAGE gel and transferred to a PVDF membrane. The membranes were blocked for 1 h and then incubated with CX3CL1 (abs155392, Absin, Shanghai, China), AKT (9272S, Cell Signaling Technology, Danvers, Massachusetts, USA), p-AKT (AF0016, Affinity Biosciences, Jiangsu, China), PIK3R5 (abs111486, Absin, Shanghai, China), PI3K (AF6241, Affinity Biosciences, Jiangsu, China), RUNX3 (27099-1-AP, Proteintech. Wuhan, China), PTEN (AF6351, Affinity Biosciences, Jiangsu, China), STAT3 (AF6294, Affinity Biosciences, Jiangsu, China), or β-Actin (66009-1-Ig, Proteintech Wuhan, China) primary antibody overnight at 4°C. After 1 h of incubation with HRP- conjugated IgG, the labeled protein was visualized using SuperSignal West Pico chemiluminescent substrate (Thermo Fisher, Waltham, MA, USA). The grayscale of immunoblots was measured by ImageJ. The protein-kinase array (R&D Systems, ARY003) was performed according to manufacturer guidelines.

Synthesis of GelMA: GelMA was synthesized according to the previous description[1]. Briefly, 20 g of gelatin (Sigma-Aldrich, St. Louis, MO) was dissolved in 200 ml of carbonate-

bicarbonate buffer (0.1 M) at 60 °C with stirring. 2 ml of methacrylic anhydride (Aladdin, Shanghai, China) was added dropwise at 0.2 ml/min to the gelatin solution and reacted for 3 h at 50 °C. The gelatin solution was then mixed with 100 ml of DPBS at 40 °C to stop the reaction. The solution was dialyzed in deionized water at 40 °C for 1 week and then the final product was freeze-dried for 3 days to form a white foam. The GelMA foam was dissolved in deuterium oxide and the chemical shifts were measured using 1H NMR (Bruker Avance NEO 400MHz, Billerica, MA,) and the degree of methacrylation was determined, which is the ratio of the number of reacted methacrylamide groups to the number of amine groups in the unreacted gelatin.

Characterization of microspheres: The morphology of microspheres was observed using an invert microscope and 100 microspheres were randomly selected for particle size analysis. The encapsulation and release of PKH26-labelled ApoVs from GA-MSP@Oxi-ApoVs were monitored at several time points (days 0, 1, 3, and 5) using an inverted fluorescence microscope. To obtain release curves of GA-MSP@Oxi-ApoVs, the protein concentration of ApoVs was initially determined using the BCA protein assay kit, and the fluorescence intensity was measured using the microplate reader (BioTek Synergy H1, Winooski, VT) to generate a standard curve. Microspheres prepared from 1 mL of GelMA-sodium alginate solution were then immersed in PBS and incubated in a horizontal shaker at 37 ± 1°C, 90 rpm for 9 days. 100 μL of the leachate was collected daily and replaced with fresh PBS. The concentration of ApoVs was obtained by detecting the fluorescence intensity of the leachate.

In vivo imaging: Fluorescence imaging analysis of living animals, MSCs or ApoVs were labeled with DiD dye (V22887, Vybrant, Thermo Fisher) following the manufacturer's instructions. MSCs or ApoVs were injection through the tail vein of nude mice. The in vivo distribution was observed on day 1, 3, and 7 by IVIS Lumina Kinetic series in vivo imaging

system with a cooled CCD camera (PerkinElmer, Waltham, MA, USA).

Histopathology and immunofluorescence staining: The collected samples were immediately stored in dry ice for frozen sections or fixed in 4% paraformaldehyde for 48 hours and then paraffin embedded. HE staining and Masson staining were performed for histological analysis in each group. ROS production at the wound site was detected using an intracellular ROS detection kit (MAK143, MAK145, Sigma-Aldrich, St. Louis, Missouri, USA). Immunostaining for CD31, α-SMA, Col I and Col III was then analyzed. The results of immunofluorescence staining for CD31 and α-SMA were calculated based on the number of new vessels in each group on days 7 and 14. Additionally, areas of neocollagen deposition in three random areas were calculated based on immunofluorescence staining for Col I/Col III using Image J software (National Institutes of Health, USA).

Figures

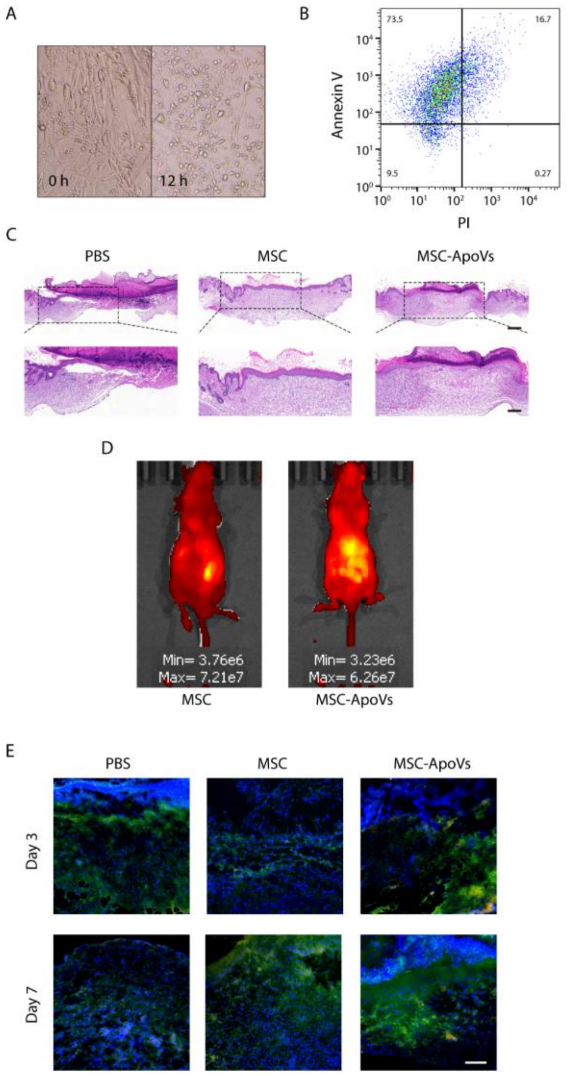

Figure S1. MSC-ApoVs identification and therapeutic efficacy in the wound microenvironment by systemic administration. (A) Bright-field microscope images before and after MSC-induced apoptosis. (B) Flow cytometry dot plots of ApoVs for Annexin V/PI costaining. (C) Typical images of HE staining on day 7 of nude mice wounds intravenously injected with MSC or MSC-ApoVs. Up: Scale bar = 400μm, down: Scale bar = 200μm. (D) Fluorescence images of frontal view of nude mice on day 1. (E) Fluorescence images of ROS staining at different time points. Scale bar = 100μm. *$p < 0.05$, **$p < 0.01$, ***$p < 0.001$ (n = 3, duplicates for each group of organisms.)

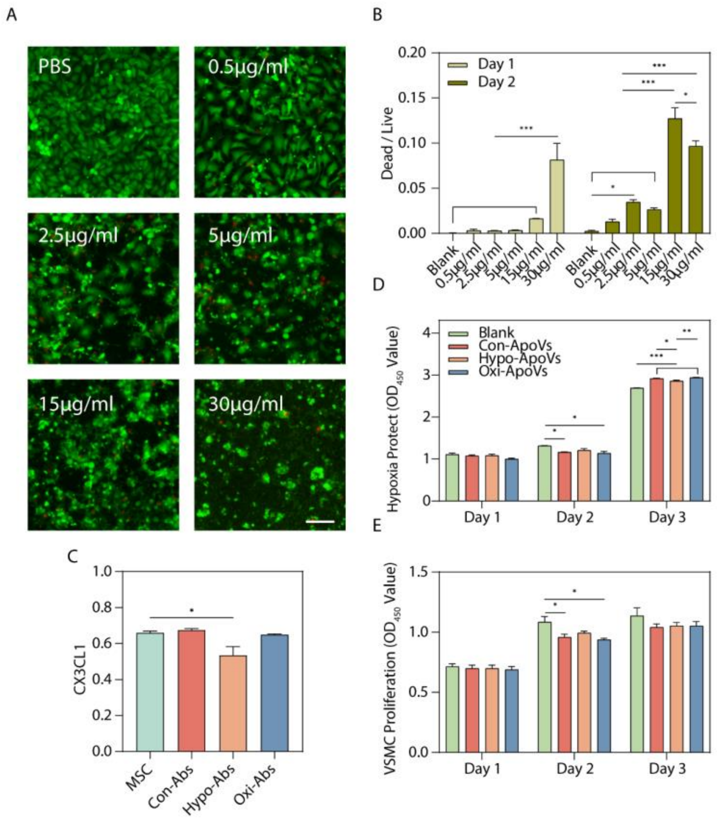

Figure S2. ApoVs biocompatibility to ECs and biofunctionality to ECs and VMSCs. (A-B) Images of live/dead staining and cell viability of ECs co-cultured with different concentrations of Con-ApoVs. Scale bar = 100μm. (C) CX3CL1 expression in Con-ApoVs, Hypo-ApoVs and Oxi-ApoVs. (D) Cell viability of ECs treated with Con-ApoVs, Hypo-ApoVs or Oxi-ApoVs under hypoxia condition. (E) Cell viability of vascular smooth muscle cells treated with Con-ApoVs, Hypo-ApoVs or Oxi-ApoVs. *p < 0.05, **p < 0.01, ***p < 0.001 (n = 3, biological replicates per group).

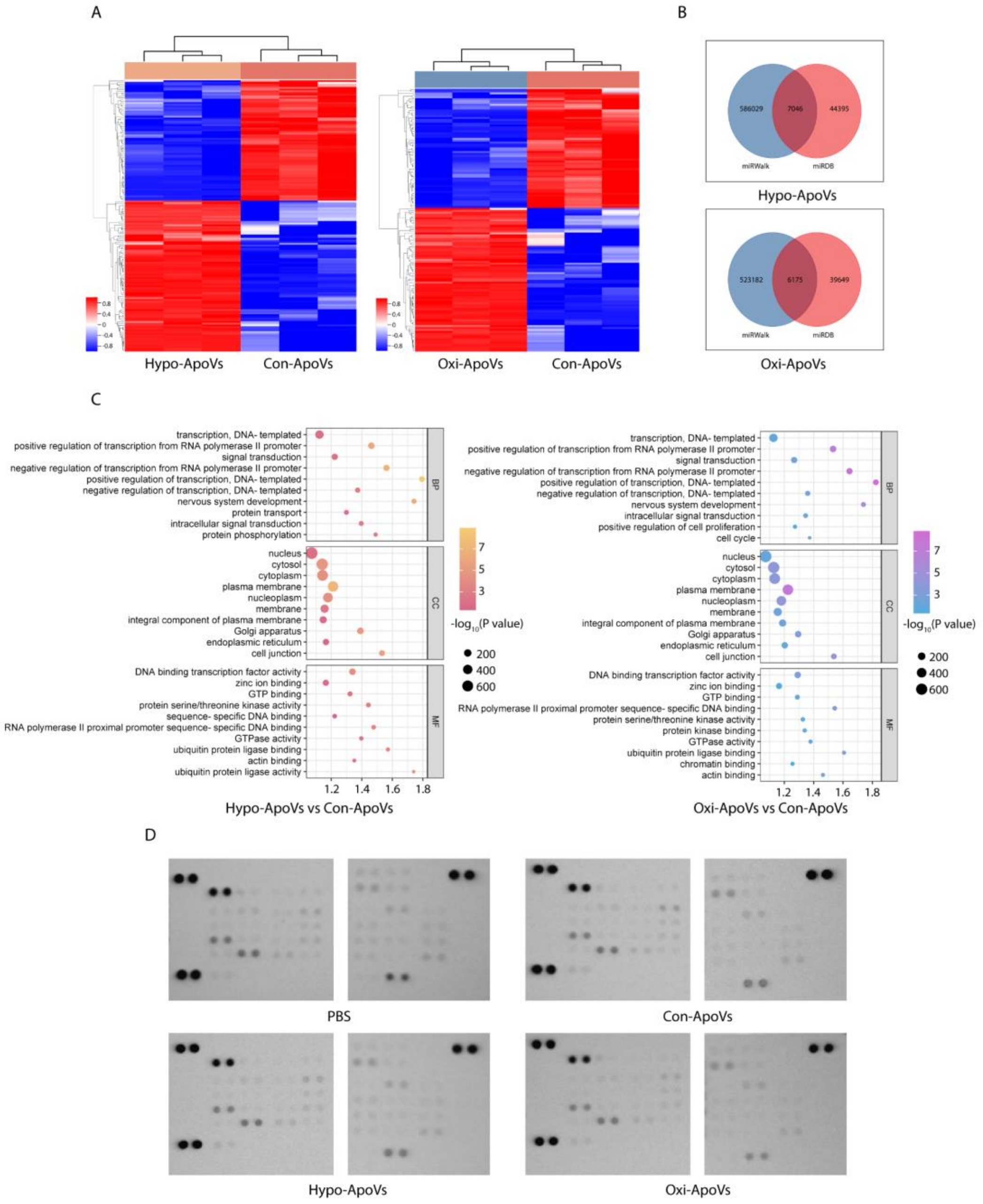

Figure S3. miRNA microarray analysis of oxygen-related environmental stressed ApoVs and ApoVs-treated EC phosphokinase microarrays. (A) Heatmap of DEmiR analysis of Hypo-ApoVs and Oxi-ApoVs compared to Con-ApoVs, respectively. (B) Wayne plots of miRNA predictions by miRDB and miRWalk databases. (C) The top 10 most significant GO entries of the three categories of Hypo-ApoVs and Oxi-ApoVs compared to Con-ApoVs, respectively. (D) Original images of phosphorylated protein arrays.

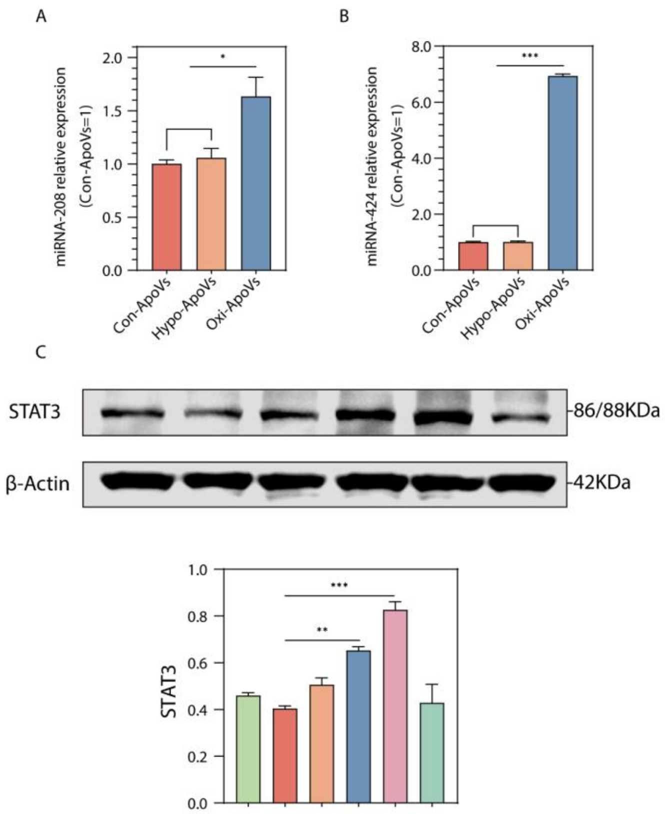

Figure S4. Differential miRNA expression in oxygen-related environmental stressed ApoVs and STAT3 expression in ECs. (A-B) qPCR analysis of miRNA-208 and miRNA-424 expression in Con-ApoVs, Hypo-ApoVs and Oxi-ApoVs. (C) STAT3 expression in ECs treated with Con-ApoVs, Hypo-ApoVs, Oxi-ApoVs, miR-Mimic or miR-Inhibitor. *p < 0.05, **p < 0.01, ***p < 0.001 (n = 3, biological replicates per group).

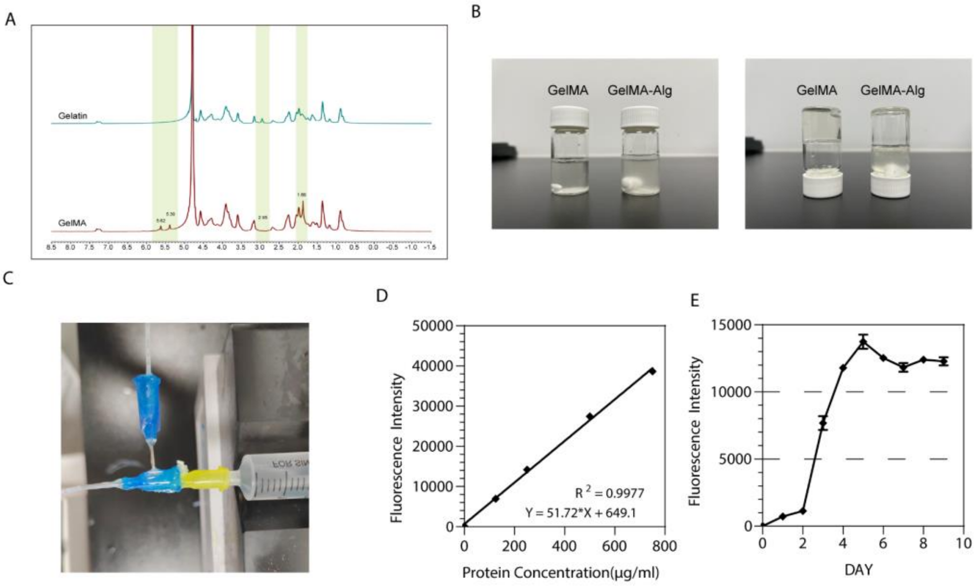

Figure S5. Characterization of GelMA-sodium alginate hydrogel microspheres loaded with Oxi-ApoVs (GA-MSP@Oxi-ApoVs). (A) ^{1}H NMR spectrum of gelatin and GelMA. (B) GelMA solution self-crosslinking at room temperature. (C) Construction of a co-axial microfluidic device for generating hydrogel droplets. (D) Standard curve of fluorescence intensity-protein concentration of PKH26-labeled Oxi-ApoVs. (E) GA-MSP@Oxi-ApoVs fluorescence release curve. *$p < 0.05$, **$p < 0.01$, ***$p < 0.001$ ($n = 3$, biological replicates per group).

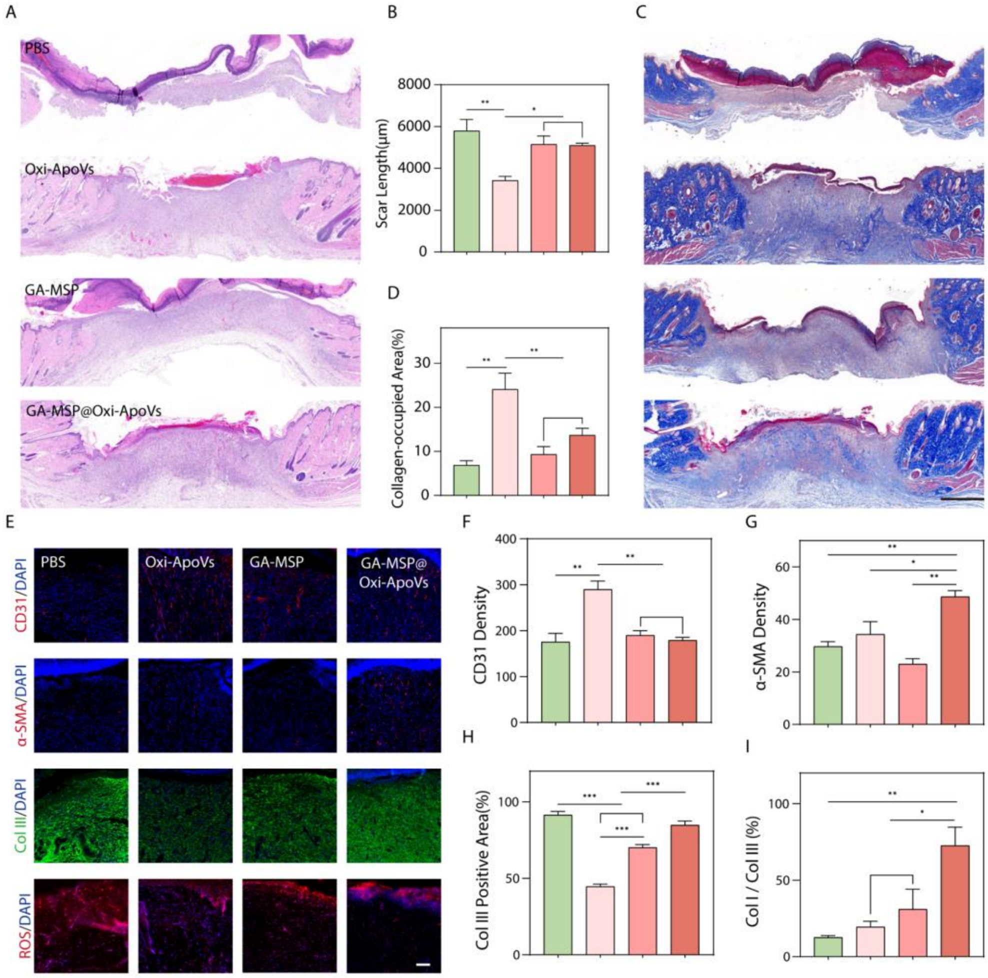

Figure. S6. Effect of local injection of GA-MSP@Oxi-ApoVs on wound healing at day 7 and at day 14. (A) Typical images of HE staining of wound tissues injected with Oxi-ApoVs, GA-MSP or GA-MSP@Oxi-ApoVs on day 7. (B) Quantitative analysis of wound length. (C) Typical images of Matson's trichrome staining on day 7. Scale bar = 1000μm. (D) Collagen deposition rate. (E) Typical images of CD31, α-SMA, COL-III, and ROS fluorescence staining on day 7. Scale bar = 100μm (F-H) CD31, α-SMA, and COL-III fluorescence density. (I) Ratio of Col I/Col III on day 14. *$p < 0.05$, **$p < 0.01$, ***$p < 0.001$ ($n = 3$, replicates per group of organisms).

Movie S1. Uniform distribution of Oxi-ApoVs in microsphere. Fluorescence videography of a GA-MSP@Oxi-ApoVs microsphere encapsulated with PKH26-labeled Oxi-ApoVs from top to bottom using an inverted fluorescence microscope showed that extracellular vesicles were uniformly distributed within the microsphere.

[1] Z. Chen, Z. Lv, Y. Zhuang, Q. Saiding, W. Yang, W. Xiong, Z. Zhang, H. Chen, W. Cui, Y. Zhang, *Adv Mater* **2023**, 35, e2300180.
